# Cholinergic Potentiation Alters Perceptual Eye Dominance Plasticity Induced by a Few Hours of Monocular Patching in Adults

**DOI:** 10.3389/fnins.2019.00022

**Published:** 2019-01-31

**Authors:** Yasha Sheynin, Mira Chamoun, Alex S. Baldwin, Pedro Rosa-Neto, Robert F. Hess, Elvire Vaucher

**Affiliations:** ^1^McGill Vision Research Unit, Department of Ophthalmology, McGill University, Montréal, QC, Canada; ^2^Laboratoire de Neurobiologie de la Cognition Visuelle, École d'Optométrie, Université de Montréal, Montréal, QC, Canada; ^3^Douglas Mental Health University Institute, McGill University, Montréal, QC, Canada

**Keywords:** neural plasticity, donepezil, neuromodulators, short-term monocular deprivation, cholinergic enhancement, ocular dominance, excitatory/inhibitory balance

## Abstract

A few hours of monocular deprivation with a diffuser eye patch temporarily strengthens the contribution of the deprived eye to binocular vision. This shift in favor of the deprived eye is characterized as a form of adult visual plasticity. Studies in animal and human models suggest that neuromodulators can enhance adult brain plasticity in general. Specifically, acetylcholine has been shown to improve certain aspects of visual function and plasticity in adulthood. We investigated whether a single administration of donepezil (a cholinesterase inhibitor) could further augment the temporary shift in perceptual eye dominance that occurs after 2 h of monocular patching. Twelve healthy adults completed two experimental sessions while taking either donepezil (5 mg, oral) or a placebo (lactose) pill. We measured perceptual eye dominance using a binocular phase combination task before and after 2 h of monocular deprivation with a diffuser eye patch. Participants in both groups demonstrated a significant shift in favor of the patched eye after monocular deprivation, however our results indicate that donepezil significantly reduces the magnitude and duration of the shift. We also investigated the possibility that donepezil reduces the amount of time needed to observe a shift in perceptual eye dominance relative to placebo control. For this experiment, seven subjects completed two sessions where we reduced the duration of deprivation to 1 h. Donepezil reduces the magnitude and duration of the patching-induced shift in perceptual eye dominance in this experiment as well. To verify whether the effects we observed using the binocular phase combination task were also observable in a different measure of sensory eye dominance, six subjects completed an identical experiment using a binocular rivalry task. These results also indicate that cholinergic enhancement impedes the shift that results from short-term deprivation. In summary, our study demonstrates that enhanced cholinergic potentiation interferes with the consolidation of the perceptual eye dominance plasticity induced by several hours of monocular deprivation.

## 1. Introduction

Changes in ocular dominance are perhaps the most widely studied form of brain plasticity, illustrating the causal links between experience and neuronal organization (Hubel and Wiesel, 1970; Wiesel, 1982; Fagiolini and Hensch, 2000; Zucker and Regehr, 2002; Bavelier et al., 2010; Gilbert and Li, 2012). Ocular dominance arises from the relative tuning of binocular neurons in the visual cortex to feedforward inputs from both eyes. Downstream competition (in the form of mutual inhibition) and integration (or binocular summation) of these monocular inputs presents an opportunity to understand the mechanisms of binocular visual processing and to explore the dynamics of experience-driven plasticity, a defining feature of the adult binocular visual system (Klink et al., 2010). A commonly used way to dissect these processes is through monocular deprivation (MD). Extended (>2 days) MD within the critical period, for instance, results in a permanent shift of perceptual eye balance in favor of the non-deprived eye that is measurable at the level of individual neurons' responses in V1 (Hubel and Wiesel, 1970; Wiesel, 1982).

In addition to plasticity during the critical period, recent investigations have also found residual plasticity in adults using short-term (a few hours) MD (Lunghi et al., 2011, 2015a,b; Hess et al., 2013; Zhou et al., 2013, 2015; Kim et al., 2017; O'Shea, 2017; for an overview of short-term MD's effects see Baldwin and Hess, 2018). In this case, patching an eye for a period of 2 h results in a temporary shift in favor of the *deprived* eye that is measurable for a duration of at least 1.5 h (Lunghi et al., 2011). Importantly, this temporary shift in perceptual eye dominance points to a latent plasticity in the adult visual system that is categorically unique from OD plasticity within the critical period because contrary to the latter, this plasticity enhances the contribution of the *deprived* eye. In an effort to avoid confusion with the classical OD plasticity examined by Hubel and Wiesel (1970), which enhances the *non-deprived* eye, we will refer to the effect examined in the present study as short-term perceptual eye dominance plasticity.

Furthermore, it is important to point out that the dissimilar effects of long-term (>2 days) and short-term MD (a few hours) could implicate a different set of neural mechanisms. In the classical model, changes in OD depend on plasticity brakes and consolidation mechanisms to modify neural activity. The short-term perceptual eye dominance plasticity observed in the present study and others (Lunghi et al., 2011; Zhou et al., 2015; Chadnova et al., 2017) is described as a form of interocular contrast gain control (Hess et al., 2013; Zhou et al., 2015), driven by enhanced contrast-gain of signal from the patched eye as well as a reduction in GABA-ergic inhibition in V1 (Lunghi et al., 2015b). Physiologically, the effects of short-term monocular deprivation have been observed using MRS (Lunghi et al., 2015b), MEG (Chadnova et al., 2017), and fMRI (Binda et al., 2017) in humans, as well as intrinsic optical imaging in a murine model (Tso et al., 2017). These studies point to deprivation-induced changes in inhibitory/excitatory dynamics in V1 with observable effects at the level of ocular dominance columns in layer 4c of V1. Importantly, frequency-tagged MEG signal from the non-deprived eye was reported to decrease during short-term deprivation and only begins recovery after restoring binocular visibility (Chadnova et al., 2017), likewise attributed to an overall enhanced net inhibition of the non-deprived eye's input relative to the deprived eye.

While mechanisms underlying neural plasticity are generally more active during development, recent investigations have demonstrated that enhanced plasticity may be restored in adulthood, albeit to a lesser degree (Bavelier et al., 2010). Treatments that enhance plasticity in adults generally do so by changing long-lasting neuronal responsiveness or by acting on so-called “brakes” on plasticity that develop after the critical period. Some of these brakes on plasticity are structural, such as peri-neuronal nets or myelin, which inhibit synaptogenesis. Others brakes are functional and act on the excitatory/inhibitory balance of neural circuits (Bear and Singer, 1986; Kasamatsu et al., 1991; Maya Vetencourt et al., 2008; Morishita et al., 2010). It is widely believed that adult brain plasticity can be enhanced by manipulating excitatory/inhibitory transmitter signaling (Bavelier et al., 2010; Morishita et al., 2010; Baroncelli et al., 2011, 2012).

Treatments that manipulate excitatory/inhibitory balance to alter neural plasticity generally act on endogenous neuromodulator activity. These interventions have, at times, been successful at enhancing cortical functioning and plasticity in both adult human and animal models (Bear and Singer, 1986; Kasamatsu et al., 1991; Bentley et al., 2003; Maya Vetencourt et al., 2008; Bavelier et al., 2010; Morishita et al., 2010; Rokem and Silver, 2010, 2013; Chamoun et al., 2017a), however this has not universally been the case (Conner et al., 2003; Chung et al., 2017). Some successful interventions targeting dopaminergic, serotonergic, and cholinergic pathways elicited direct consequences on adult functional and structural brain reorganization (Bear and Singer, 1986; Berardi et al., 2000; Bao et al., 2001; Weinberger, 2007; Maya Vetencourt et al., 2008; Morishita et al., 2010).

Of the known neuromodulators, acetylcholine (ACh) is particularly interesting for visual plasticity because of its role in modulating excitatory/inhibitory balance in visual cortex as well as mediating long-lasting neuronal responsiveness and structural plasticity throughout the cortex. For instance, genetically removing the expression of Lynx1, a cholinergic brake, reinstates critical-period-like OD plasticity in adult mice (Morishita et al., 2010), where the non-deprived eye becomes more dominant. Furthermore, multiple administrations of the acetylcholinesterase inhibitor (AChEI) physostigmine (which potentiates and prolonges the action of endogeneous ACh) improves visual function and enhances critical-period-like ocular dominance plasticity after long-term MD in a murine model of amblyopia (Gagolewicz and Dringenberg, 2009; Morishita et al., 2010; Groleau et al., 2015).

In humans, drugs that increase endogenous ACh signaling have been shown to enhance cortical plasticity and functioning by refining neural circuits' efficacy and enhancing perceptual learning. This has been assessed, for example, in visual tasks such as motion direction discrimination (Silver et al., 2008; Rokem and Silver, 2010, 2013), and 3D multiple object tracking (Chamoun et al., 2017a). There are other instances, however, that demonstrate the opposite: a recent study reported that cholinergic enhancement blocked the effect of perceptual learning of a crowding task relative to a placebo control group (Chung et al., 2017). Nevertheless, pharmacological enhancement of synaptic ACh has been shown to improve visual function, possibly by reducing the spatial spread of visual responses, sharpening visual spatial perception, increasing top-down control of attentional orienting and stimulus discrimination, and enhancing cortical activation in V1 (Silver et al., 2008; Klinkenberg et al., 2011; Kang et al., 2014; Gratton et al., 2017). Although cholinergic potentiation has been implicated in mediating several types of visual perceptual learning and enhancing visual neural responsiveness, its exact role in adult visual plasticity *per se* remains unclear.

In the present study, we used the AChEI donepezil to investigate the effects of cholinergic enhancement on adult perceptual eye dominance plasticity. In a double-blind crossoever design, we provided a placebo pill or donepezil and compared the effect of a few hours monocular patching on perceptual eye dominance in the two experimental conditions. Under the assumption that cholinergic potentiation enhances visual neural responsiveness (Kang et al., 2014), we hypothesized that donepezil would (1) enhance the strength of the patched eye's contribution to binocular vision after patching and would also (2) reduce the amount of time necessary to elicit the shift in perceptual eye dominance relative to the placebo control. We were surprised to find that donepezil in fact *reduces* both the magnitude and duration of the shift of binocular response in favor of the deprived eye. We found this to be the case when patching for both 1 and 2 h, and with two separate tasks measuring perceptual dominance.

## 2. Materials and Methods

Sixteen young adults participated in the study. Two participants were excluded from the study due to data collection errors. One additional subject was excluded from analysis on the basis that he was an author and aware of the motivation of the investigation. Thirteen participants (2 Men, age: 19–31 years, BMI: 18–26 kg/m^2^, see Table 1) completed the study. Twelve subjects completed the first experiment which used the binocular phase combination task to measure perceptual eye dominance before and after 2 h of deprivation. Seven participants completed the second experiment which also used the phase combination task to measure perceptual eye dominance after 1 h of deprivation. Finally, six participants completed a different experiment which used binocular rivalry to measure the shift in perceptual eye dominance after 2 h of monocular deprivation. Only two subjects were able to participate in all three experiments.

**Table 1:** Demographic data: participant characteristics and involvement in binocular combination and binocular rivalry experiments, mean ± SEM (range).

**Experiment**	***N***	**Age**	**Height(cm)**	**Weight (kg)**	**BMI (kg/m)**
Total	13	23 ± 1 (19–31)	169 ± 4 (152–193)	66 ± 2 (43–77)	23 ± 1 (18–26)
BPC2	12	23 ± 1 (19–31)	170 ± 1 (152–193)	64 ± 1 (43–90)	22 ± 1 (18–26)
BPC1	7	25 ± 1 (20–31)	174 ± 2 (158–193)	68 ± 2 (56–90)	22 ± 1 (19–24)
RIV2	6	24 ± 1 (20–28)	171 ± 2 (152–193)	62 ± 2 (43–90)	21 ± 1 (18–24)

*BPC2: Binocular Phase Combination Task—2 h monocular deprivation; BPC1: Binocular Phase Combination Task—1 h monocular deprivation; RIV2: Binocular Rivalry Task—2 h monocular deprivation*.

All subjects met the inclusion criteria (Table 2). The body-mass-index range was specified as 17–26 kg/m^2^ to ensure a similar distribution of the drug across subjects. All subjects were naive to the purpose of the experiment. A standard clinical and neurological examination, a stereoacuity test and an ECG recording were performed before the beginning of the experiment. Subjects were monitored for their safety during the experimental sessions with several blood pressure measurements taken.

**Table 2:** Inclusion and exclusion criteria.

**Inclusion criteria**	**Exclusion criteria**
Good health	Attention deficit
Body mass index between 17 and 26	Smoker
No visual impairment or ocular pathology not corrected by glasses or contact lenses	Pregnant, breast feeding, or planning a pregnancy
Good stereo vision	Unable to do task
	Lactose intolerant (lactose pills as placebo)

Subjects gave written informed consent prior to the experiment. Data were collected and kept secure in the laboratory of author EV. Participants were enrolled by the student researcher YS, and their random allocation sequence was carried out by EV and MC by assigning drug/placebo in numbered containers. Subjects received financial compensation to cover travel expenses and time spent participating in the experiment at a rate of $15/h. The procedures were in accordance with the Helsinki Declaration of 2013 and the ethical standards of the Comité d'éthique de la recherche en santé, Université de Montréal, approval #12- 084-CERES-P.

We used a double-blind within-subject crossover design where each participant completed two experimental sessions. Subjects were randomly assigned to either the donepezil or control group for their first session and then switched to the other group for their second session which occurred 21 days after the first. In each session, participants completed baseline testing on either a binocular phase combination or binocular rivalry task. This provided an index of their baseline perceptual eye dominance. This was followed by donepezil or placebo administration and 2 h of monocular deprivation. The patch was then removed, and subsequent tests of perceptual eye dominance were made over the next hour.

In experiment two, subjects underwent an identical protocol to that of experiment one, with the exception of adjusting the incubation period to 2 h and the deprivation duration to 1 h. A third experiment was also conducted where a binocular rivalry task was used instead of a binocular combination task. Previous studies on short-term monocular deprivation have found different results with the two tasks (Bai et al., 2017)—depriving one eye of Fourier phase information for 2 h produced a shift in perceptual eye dominance in favor of the deprived eye as measured with binocular rivalry but not with binocular phase combination. We were interested to determine whether donepezil had a different effect on plasticity as measured through rivalry vs. binocular phase combination.

### 2.1. Donepezil Pharmacological Enhancement

Donepezil is a reversible, non-competitive, highly selective AChEI with a half-life of 80 h and a peak plasma level of 4.1 ± 1.5 h after intake (Rogers and Friedhoff, 1998). Five milligrams of donepezil is the lowest prescribed dose which induces beneficial cognitive effects with very low adverse reaction incidence (Prvulovic and Schneider, 2014). This dose is shown to be effective in improving visual attention and the neural plasticity associated with perceptual learning in young adults (Rokem and Silver, 2010, 2013). Three hours before the patch removal, subjects ingested one capsule containing either 5 mg donepezil (ARICEPT, Pfizer, Canada) or lactose placebo with water (Rokem and Silver, 2010). The experimenter and subjects were naive to the experimental conditions.

### 2.2. Monocular Deprivation

Using the Miles test for sensory eye dominance (Miles, 1930), we identified the dominant eye for each participant. We then patched the *non*-dominant eye for each experimental session they participated in. We chose to patch the non-dominant eye with the rationale that it has more capacity to increase its dominance, however this has not been yet been assessed systematically. We used a diffuser eye patch that preserved most luminance information (40% luminance reduction), but eliminated all spatial frequency information as confirmed by a Fourier decomposition of a natural image viewed through the patch. While most studies use a patching duration of 2.5 h, recent investigations have seen comparable effects after 2 h of patching (Lunghi et al., 2016). To minimize the amount of time it would take to complete a single session, we administered monocular deprivation for 2 h for Experiments 1 and 3. For Experiment 2 we reduced the duration to 1 h to assess whether donepezil accelerates the rate of plasticity.

### 2.3. Experimental Protocol

**Figure 1:**
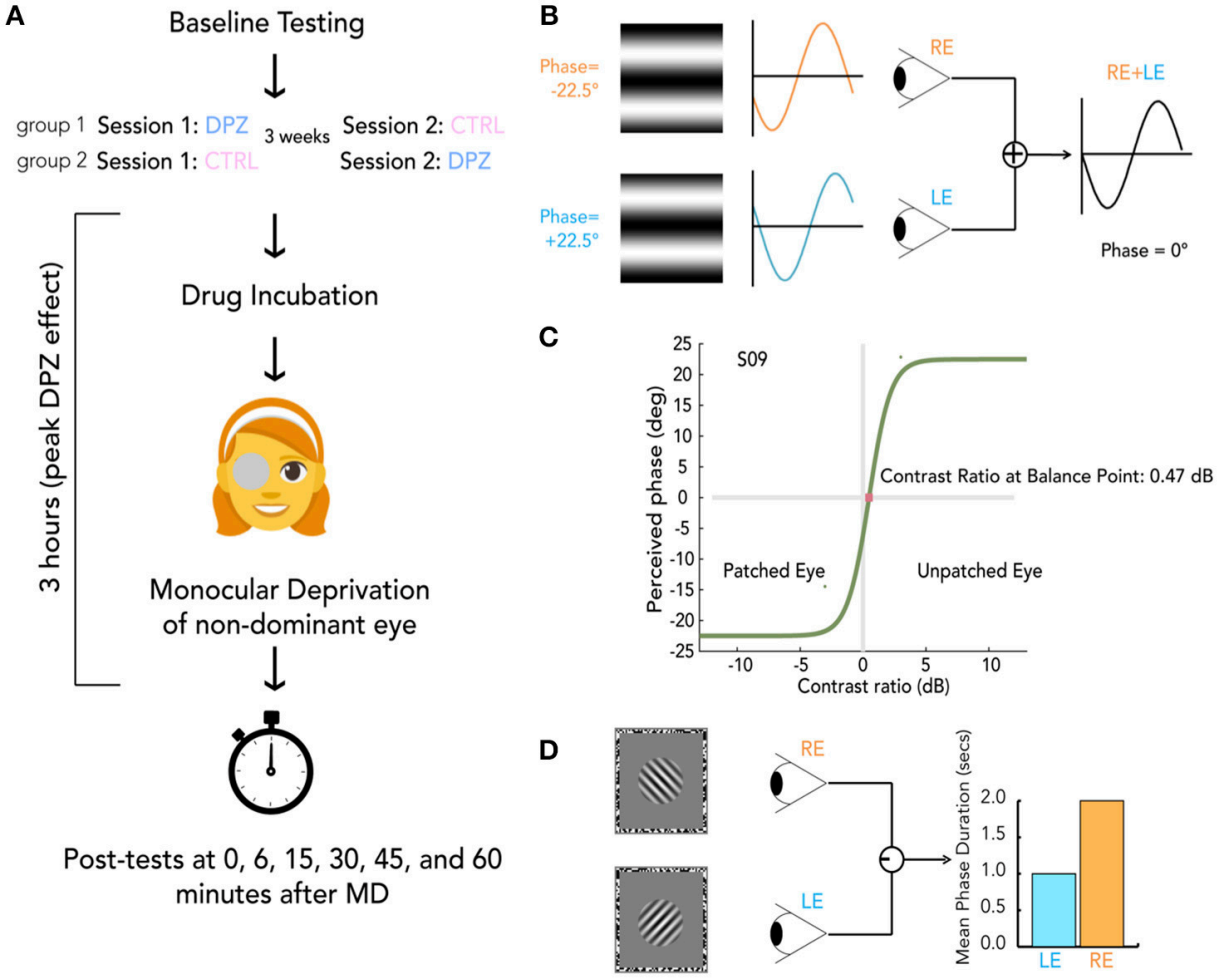
General protocol and methods. **(A)** Schema of experimental session. For each experiment, participants were randomly allocated to either group 1 or group 2, indicating whether they take donepezil (DPZ) or placebo on the first day and then the reverse on the second day which occurs 21 days later. After baseline testing, participants take their assigned pill. Both the experimenter and participant are unaware of the participant's group assignment. After a drug incubation period (1 h for experiments 1 and 3, 2 h for experiment 2), Monocular deprivation (MD) with a diffuser eye patch begins (2 h for experiments 1 and 3, 1 h for experiment 2). Post-MD testing begins 3 h after taking the pill. **(B)** Binocular phase combination task. The participant views two sinusoidal gratings presented individually to each eye through a modified Wheatstone stereoscope. The gratings have phase-shifts in opposite directions of the same magnitude (22.5°). The observer is asked to use keypresses to move a flanking bar to the middle of the trough of the fused sinusoid. This gives an estimate of the perceived phase of the grating after binocular combination. In this example, the participant sees a fully balanced fusion of the two gratings, resulting in a perceived phase difference of 0°. **(C)** Psychometric curve for binocular combination task. Psychometric function for one subject at baseline. Curves were generated by fitting data from each measurement to a model of binocular combination (see section Materials and Methods). The CR at the balance point was used to determine ocular perceptual eye dominance for each measurement. **(D)** Binocular rivalry task. Two orthogonal sinusoidal gratings ± 45° were presented dichoptically through a modified Wheatstone stereoscope for 180 s per measurement. The participant continuously indicated whether they were seeing a (1) predominantly left-tilted grating, (2) a balanced fusion of right and left lines, or (3) a predominantly right-tilted grating for the entire duration of the stimulus presentation. The ratio of median rivalry phase durations for each eye was used to quantify ocular dominance for each measurement.

The general protocol of each session is outlined in Figure 1A. For each of the three experiments, participants were randomly allocated to either Group 1 (Donepezil first session, Placebo second session) or Group 2 (Placebo first session, Donepezil second session). The experimenter was not aware of participants' group assignments until after data collection was complete. For safety purposes, the experimenter recorded the participant's systolic blood pressure at baseline and monitored blood pressure levels throughout the experiment. Baseline psychophysical testing took place over the course of 5–10 min on either the binocular phase combination task in Experiments 1 and 2 or binocular rivalry in Experiment 3. The half-life of donepezil is 4.1 ± 1.5 h after intake. We therefore chose to begin post-deprivation testing at 3 h after drug administration to maximize the potency of the drug at the time of testing. For Experiments 1 and 3 this required that our participants wait 1 h before beginning their 2 h of deprivation. For Experiment 2 participants waited 2 h before beginning 1 h of deprivation.

After the drug incubation period, participants were provided with a diffuser eye patch to wear on the non-dominant eye. During the drug incubation period and subsequent monocular deprivation, participants were instructed to keep their eyes open and do activities that require visual perception such as watching a movie, doing homework, or walking around the lab.

After the full duration of monocular deprivation (2 h for Experiment 1 and 3, 1 h for Experiment 2), participants were instructed to remove the eyepatch and begin psychophysical testing. Psychophysical measurements were taken at five timepoints (0, 15, 30, 45, and 60 min) after deprivation. Each measurement took 3 min to complete, and participants were instructed to keep their eyes open in between measurements. After completing the first session of an experiment, participant were assigned a scheduled date to return for completing their second session. To ensure there was no residual effects from the previous session, all sessions were spaced roughly 3 weeks apart from one another.

#### 2.3.1. Apparatus

Each session took place in a quiet room with dim light. Visual stimuli for both binocular combination and binocular rivalry experiments were generated and controlled by an Apple MacBook Pro 2008 computer (MacOSX; Cupertino, CA, USA) running MATLAB R2012B (MathWorks, Natick, MA) with the Psychtoolbox psychophysics toolbox (Brainard, 1997; Pelli, 1997). Stimuli were presented on a gamma-corrected cathode ray tube monitor (LG, Seoul, South Korea) driven at a resolution of 1,024 × 768 pixels, with a refresh rate of 75 Hz and a measured mean luminance of 60 cdm^−2^. Participants viewed stimuli through an eight-mirror modified Wheatstone stereoscope so that the left image was only seen by the left eye and the right image by the right eye. The position of the participant's head was stabilized with a chin rest at a viewing distance of 57 cm.

#### 2.3.2. Binocular Phase Combination Task

The binocular phase combination task (Ding and Sperling, 2006) is outlined in Figure 1B. Each measurement began with a dichoptic nonius cross presented inside a 3° oval surrounded by a black-and-white noise (1 cycle per degree) frame (side = 10°). The observer was asked to make keypresses to adjust the position of the two frames to calibrate the optimal position for comfortable fusion. After calibration, two horizontal sine-wave gratings (0.3 cycles per degree, 6° × 6°) with phase-shifts in opposite directions of the same magnitude (22.5°) were presented dichoptically through the stereoscope.

The physical sum of two sinusoidal gratings of the same frequency is another sinusoidal grating with a phase and amplitude that depend on the phases and amplitudes of the two inputs. This behavior has also been shown to hold for the perception that arises from the summation of gratings presented to the two eyes (Ding and Sperling, 2006). For our stimuli, the perceived phase of the perceived grating depends on the internal weighting of the inputs from each eye. Therefore, variations in perceptual eye dominance can be quantified by the change in the perceived phase (Figure 1B).

To account for any potential bias, two configurations were used for assessing the perceived phase in any given trial. The first configuration gave a phase-shift of +22.5° in the dominant eye and −22.5° in the non-dominant eye. The second reversed the two, giving a phase-shift of −22.5° in the dominant eye and +22.5° in the non-dominant eye. In each trial, participants were asked to indicate the location of the central dark bar of the fused grating by adjusting the location of a flanking bar on the screen with a keyboard. The vertical position of the flanking bar was converted into degrees of phase of the combined gratings. This phase offset provided a subjective measure of perceived phase in each trial. An increase of the perceived phase (i.e., more positive) after deprivation indicates an enhanced contribution of the eye that was not patched, whereas a decrease of the perceived phase (i.e., more negative) indicates a shift of dominance toward the patched eye. After each trial, the nonius calibration screen was presented for the observer to re-calibrate if necessary and begin the next trial.

To fit our data to psychometric curves defined by a model of binocular combination (Ding and Sperling, 2006; Huang et al., 2009), we modulated the interocular contrast ratio around a mean contrast of 50% across trials (Figure 1C). For baseline measurements, each of the following ratios were tested eight times by method of constant stimuli: 1:2, 1:$\sqrt{2}$,1:1, $\sqrt{2}$:1, 2:1. Due to the time-sensitive nature of OD plasticity after removing the eye-patch, post-test measurements were reduced to three ratios: 1:$\sqrt{2}$,1:1, $\sqrt{2}:1$. Baseline data consisted of perceived phases collected from 80 trials (5 contrast ratios × 8 repetitions × 2 configurations), and post-deprivation measures consisted of perceived phases from 30 trials (3 contrast ratios × 5 repetitions × 2 configurations). Data were fit to a function of the form

(1) $$\Phi_{A}=2\tan^{-1}\left[\frac{f(\alpha ,\delta ,\gamma )-\delta^{1+\gamma }}{f(\alpha ,\delta ,\gamma )+\delta^{1+\gamma }}\tan\left(\frac{\theta }{2}\right)\right],$$

where

(2) $$f\left(\alpha ,\delta ,\gamma \right)=\frac{1+\delta^{\gamma }}{1+\alpha \delta^{\gamma }},$$

and Φ_*A*_ is the perceived phase of the fused image, θ is the constant phase displacement between eyes (45°), δ is the interocular contrast ratio, and the two free parameters, γ and α are the slope of the function and the contrast ratio when the two eyes contribute equally to binocular vision. α is represented in log units (dB relative to a 1:1 contrast ratio between the two eyes), calculated as

(3) $$\alpha_{\text{dB}}=20\times \log_{10}\left(\delta_{\text{balanced}}\right)$$

such that an α of 0 dB indicates that both eyes are contributing equally to binocular combination, while an α of −6 dB indicates that input from the deprived eye is weighted roughly twice as much as that from the non-deprived eye. Changes in α provide a measure of the shift in perceptual eye dominance from baseline. Our main measure of deprivation-induced changes in dominance as measured by the binocular phase combination task was obtained by subtracting each participant's baseline α from each post-patching α.

#### 2.3.3. Binocular Rivalry

In Experiment 3 subjects performed a binocular rivalry task (see Figure 1D) instead of a phase combination task. After calibration (as above), two orthogonal (± 45°) sinusoidal gratings (3 cycles per degree, subtending a diameter of 1.5°, with a raised cosine annulus blurring the edges, contrast = 75%) were presented inside a black-and-white noise pattern frame (1 cycle per degree, 10 °, one side) individually to each eye. The participant was asked to continuously indicate whether they were seeing a (1) predominantly left-tilted grating, (2) a balanced fusion of right and left tilted gratings, or (3) a predominantly right-tilted grating. Baseline measurements were made from six 90-s rivalry blocks. Each post-patching measure was made using two 90-s rivalry blocks.

A commonly used measure of perceptual eye dominance when analysing rivalry data is the mean phase duration (Lunghi et al., 2011, 2015b, 2016). This measure is defined as the average amount of time spent viewing a percept by one eye. Rivalry phase durations generally follow a log-normal distribution (Zhou et al., 2004). Because of this property, mean phase durations are generally influenced more by longer phase durations. The median phase duration is arguably a better measure of centrality for these distributions, so our analysis used the median phase durations to compute an perceptual ocular dominance index (ODI), bounded by [−1, 1], for each rivalry measurement that was defined by the equation:

(4) $$\text{ODI}=\left(\frac{{\bar{d}}_{\text{non-deprived}}-{\bar{d}}_{\text{deprived}}}{{\bar{d}}_{\text{non-deprived}}+{\bar{d}}_{\text{deprived}}}\right),$$

where the two $\bar{d}$ variables are the mean phase durations for the non-deprived and deprived eyes. Negative and positive ODIs indicate bias in favor of the deprived and non-deprived eyes, respectively. To evaluate deprivation-induced changes in the index we then subtracted baseline values from each post-patching measure.

###  Statistical Analyses

Each experiment provided measures of perceptual eye dominance at baseline and at five time points (0, 15, 30, 45, 60 min) after treatment. For our analyses, we subtracted baseline ODIs from each post-deprivation ODI to obtain five treatment-induced differences in perceptual eye dominance over the course of an hour after removing the patch. We implemented a two-factor (treatment × time) repeated measures ANOVA on these post-baseline differences to investigate whether there was an interaction between the donepezil and placebo control treatments over the course of our measurements. Separately, we applied one-way repeated measure ANOVAs for each treatment condition to determine whether treatment significantly shifted perceptual eye dominance at the initial time point after patching with respect to baseline. The results of our ANOVA analyses for the three experiments are summarized in Table 3. If the effect of treatment was significant for either of the experimental conditions, we conducted follow-up FDR-corrected (Benajmini and Hochberg, 1995) *t*-tests on the post-baseline differences to determine which time points were significantly shifted with respect to baseline. In addition, we computed the area under the curve generated by drawing a line through each treatments's post-baseline differences as a function of time. This measure, calculated by estimating the integral (via the trapezoidal method) of the curve, can be used as an estimate of the overall effect size for each treatment (donepezil or placebo). We applied Wilcoxon signed-rank tests on these areas to determine if there were significant differences in (1) the mean ranks of the areas from those at baseline and (2) between the mean ranks of the areas of the two treatment conditions. All data used for the statistical analysis have been made available online in the Supplementary Material associated with this article.

**Table 3:** ANOVA summary table.

	**Donepezil vs. placebo**						**Initial effect of treatment**					
	**Source**	**df**	**MS**	**F**	***p***	**$\eta_{p}^{2}$**	**Source**	**df**	**MS**	**F**	***p***	**$\eta_{p}^{2}$**
BPC2	Session	1	48.50	11.10	0.00^*^	0.50	DPZ	1	13.50	10.63	0.00^*^	0.49
	Time	4	6.62	4.60	0.00^*^	0.30	CTRL	1	56.20	38.30	0.00^*^	0.77
	Session × Time	4	1.25	0.83	0.51	0.07						
BPC1	Session	1	7.90	5.40	**0.06**	0.47	DPZ	1	4.36	6.70	0.04^*^	0.46
	Time	4	2.67	7.80	0.00^*^	0.50	CTRL	1	7.00	17.20	0.00^*^	0.74
	Session × Time	4	0.53	0.91	0.47	0.13						
RIV2	Session	1	0.03	1.19	0.32	0.19	DPZ	1	0.08	0.91	0.38	0.15
	Time	4	0.10	3.32	0.03^*^	0.40	CTRL	1	0.25	8.00	0.03^*^	0.61
	Session × Time	4	0.01	1.41	0.28	0.35						

*The left column shows the results of a two-factor (session × time) repeated-measures ANOVA for the three separate experiments. The right column shows results from one-factor (time) repeated measures ANOVAs conducted for individual sessions (donepezil/control) for each experiment to determine whether the effect of treatment was significantly different from that measured at baseline. BPC2: Binocular Phase Combination Task—2 h monocular deprivation; BPC1: Binocular Phase Combination Task—1 h monocular deprivation; RIV2: Binocular Rivalry Task—2 h monocular deprivation. Asterisks indicate statistically significant effects, p < 0.05*.

## 3. Results

###  Experiment 1: 2 h of MD With Binocular Phase Combination Task

**Figure 2:**
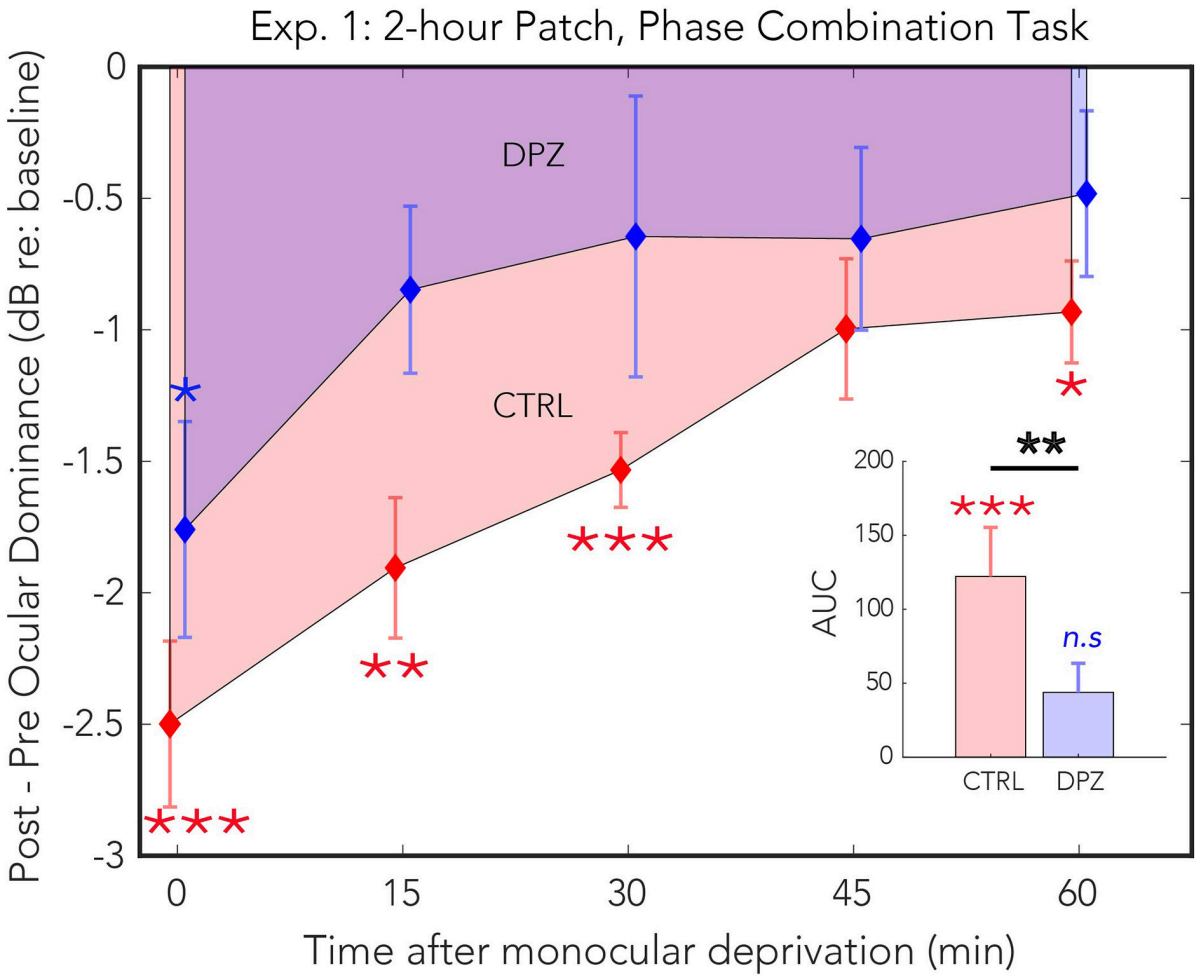
Experiment 1: The effect of donepezil on the shift in ocular dominance that occurs after 2 h of monocular deprivation, measured by binocular phase combination. Donepezil reduces both the magnitude and the duration of the shift in perceptual eye dominance that results from monocular deprivation relative to placebo control. *N* = 12. Red and blue diamonds indicate the mean difference in ocular dominance from that measured at baseline using the contrast ratio index described in Equation (3) for control (CTRL) and donepezil (DPZ) conditions. Errorbars are bootstrapped SEMs. Red and blue asterisks indicate means that are significantly different from baseline for CTRL and DPZ conditions, respectively. Black asterisks indicate means that are significantly different from one another. ^***^FDR-corrected *p* < 0.001, ^**^FDR-corrected *p* < 0.01, ^*^FDR-corrected *p* < 0.05.

Two hours of patching induced a shift in perceptual dominance for both donepezil and placebo control conditions [CTRL: Wilks' lambda = 0.22, *F*_(1, 11)_ = 38.3, FDR-corrected *p* < 0.01, $\eta_{p}^{2}$ = 0.77; DPZ: Wilks' lambda = 0.50, *F*_(1, 11)_ = 10.63, FDR-corrected *p* < 0.05, $\eta_{p}^{2}$ = 0.49] that was maximal immediately after removing the patch (CTRL: *M* = −3.06, 95% CI: [−4.1, −1.9]; DPZ: *M* = −1.5, 95% CI: [−2.5, −0.48], dB with respect to baseline). We performed a two-factor (session × time) repeated measures ANOVA (see Table 3 for statistics) on the post-baseline ODIs computed for measurements taken at 0, 15, 30, 45, and 60 min after removing the patch. The results of this analysis yielded significant main effects for both session [Wilks' lambda = 0.5, *F*_(1, 11)_ = 11.1, *p* < 0.01, $\eta_{p}^{2}$ = 0.50] and time [Wilks' lambda = 0.08, *F*_(4, 44)_ = 4.6, *p* < 0.01, $\eta_{p}^{2}$ = 0.30], however the interaction term was not significant [*F*_(4, 44)_ = 0.83, *p* > 0.05] (Figure 2).

A *post hoc* paired *t*-test examining the main effect of session indicated that the mean post-baseline difference across all measured time points observed when subjects were treated with donepezil was significantly reduced relative to the placebo control condition [*t*_(11)_ = −4.9, *p* < 0.001, *M* = −1.27, 95% CI: [−1.79, −0.75]]. Subsequent FDR-corrected paired *t*-tests on the post-baseline ODIs in the donepezil condition indicated that the mean shift in perceptual eye dominance was significant only during the first measured time point after removing the patch [*t*_(11)_ = −3.2, FDR-corrected *p* < 0.05]. No other measured time points in the donepezil condition were significantly shifted from baseline (FDR-corrected ps < 0.05). Post-baseline differences in the placebo control condition, on the other hand, remained significant until up to at least 60 min after removing the patch (FDR-corrected ps < 0.05), indicating that donepezil significantly reduced the duration that the mean shift in perceptual eye dominance was significantly shifted from baseline compared to the placebo control.

Furthermore, a two-tailed paired Wilcoxon signed-rank test on the mean ranks of the areas under the curves generated by the post-baseline ODIs in each condition revealed that the mean rank area observed in the placebo control condition was significantly greater than zero (FDR-corrected *p* < 0.001), while the mean rank area observed in the donepezil condition was not significantly different from zero (FDR-corrected *p* > 0.05). An additional signed-rank test on the mean ranks of the areas of the two experimental conditions revealed that the mean rank area observed in the donepezil condition was significantly reduced relative to placebo control (*p* < 0.01), demonstrating that the magnitude of the effect of patching treatment across the five measured time points was significantly reduced in the donepezil condition compared to placebo. Together, the results of these analyses indicate that donepezil significantly reduces the magnitude and duration of the shift in perceptual eye dominance that occurs after 2 h of monocular deprivation.

### 3.1. Experiment 2: 1 h of MD With Binocular Phase Combination Task

**Figure 3:**
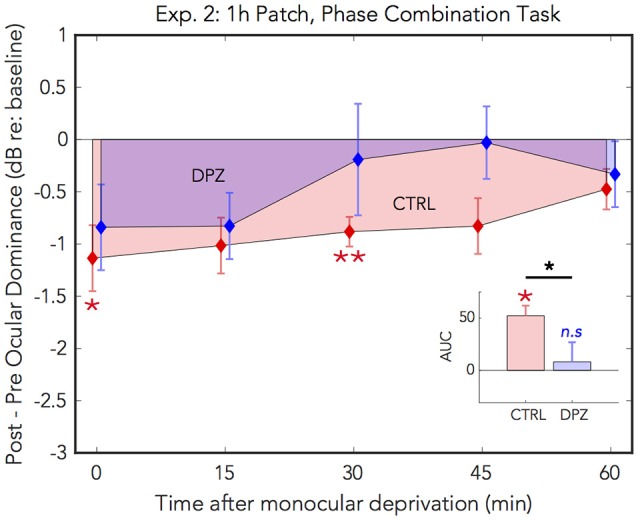
Experiment 2: The effect of donepezil on the shift in perceptual eye dominance that occurs after 1 h of monocular deprivation, measured by binocular phase combination. Donepezil reduces the magnitude and duration of the shift in ocular dominance induced by 1 h of monocular patching. *N* = 7. For further details see Figure 2 caption.

As in the first experiment, 1 h of patching induced a shift in perceptual eye dominance for both donepezil and placebo control conditions [CTRL: Wilks' lambda = 0.26, *F*_(1, 6)_ = 17.2, FDR-corrected *p* < 0.01, $\eta_{p}^{2}$ = 0.74; DPZ: Wilks' lambda = 0.47, *F*_(1, 6)_ = 6.7, FDR-corrected *p* < 0.05, $\eta_{p}^{2}$ = 0.52] that was maximal immediately after removing the patch (CTRL: *M* = −1.4, 95% CI: [−2.3, −0.6]; DPZ: *M* = −1.1, 95% CI: [−2.1, −0.06], dB with respect to baseline). We performed a two-factor (session × time) repeated measures ANOVA (see Table 3 for statistics) on the post-baseline ODIs computed for measurements taken at 0, 15, 30, 45, and 60 min after removing the patch. The results of this analysis yielded a significant main effect of time [Wilks' lambda = 0.10, *F*_(4, 24)_ = 7.8, *p* < 0.001, $\eta_{p}^{2}$ = 0.57], and a trend toward a significant main effect of session [Wilks' lambda = 0.53, *F*_(1, 6)_ = 5.4, *p* = 0.06, $\eta_{p}^{2}$ = 0.47], however the interaction term was not significant [*F*_(4, 24)_ = 0.9, *p* > 0.05] (Figure 3)

*Post hoc* paired *t-*tests examining the main effect of session yielded a trend that the mean post-baseline difference observed when subjects were treated with donepezil was reduced relative to the placebo control (*M* = −0.66, *p* = 0.06, 95% CI: [−1.4, 0.03]). Subsequent FDR-corrected paired *t-*tests on the post-baseline ODIs indicated that the mean shift in perceptual eye dominance was was significant at 0 and 30 min after patching in the placebo control condition (FDR-corrected ps < 0.05), however no individual time points were significantly shifted from baseline in the donepezil condition (FDR-adjusted *p* > 0.05). As in experiment 1, this indicates that donepezil reduced the duration that perceptual eye dominance was shifted from baseline compared to the placebo control.

A two-tailed paired Wilcoxon signed-rank test on the mean ranks of the areas under the curves generated by the post-baseline ODIs in each condition revealed that the mean rank area observed in the placebo control condition was significantly greater than zero (FDR-corrected p < 0.05), while the mean rank area observed in the donepezil condition was not significantly different from zero (FDR-corrected *p* > 0.05). Likewise, an additional Wilcoxon signed-rank test on the mean ranks of the areas in the two experimental conditions revealed that the mean rank area of the donepezil condition was reduced relative to the placebo control (*p* < 0.05), further demonstrating that the overall magnitude of the effect of 1 h of patching on perceptual eye dominance was reduced in the donepezil condition. Together, the results of these analyses indicate that donepezil significantly reduces the magnitude and duration of the shift in perceptual eye dominance that occurs after 1 h of deprivation.

### 3.2. Experiment 3: 2 h of MD With Binocular Rivalry

For experiment 3, we used a binocular rivalry task to measure perceptual eye dominance before and after 2 h of monocular deprivation. We administered a repeated measures ANOVA on the initial ODI measured after deprivation vs. baseline to assess the initial effect of patching in the two experimental conditions. Ocular dominance shifted significantly with respect to baseline in the placebo control condition, however not in the donepezil condition [CTRL: Wilks' lambda = 0.38, *F*_(1, 5)_ = 7.91, FDR-corrected *p* < 0.05, $\eta_{p}^{2}$ = 0.61, *M* = −0.29, 95% CI: [−0.55, −0.30]; DPZ: Wilks' lambda = 0.85, *F*_(1, 6)_ = 0.91, FDR-corrected *p* = 0.38, $\eta_{p}^{2}$ = 0.15, *M* = −0.16, 95% CI: [−0.59, 0.27]].

**Figure 4:**
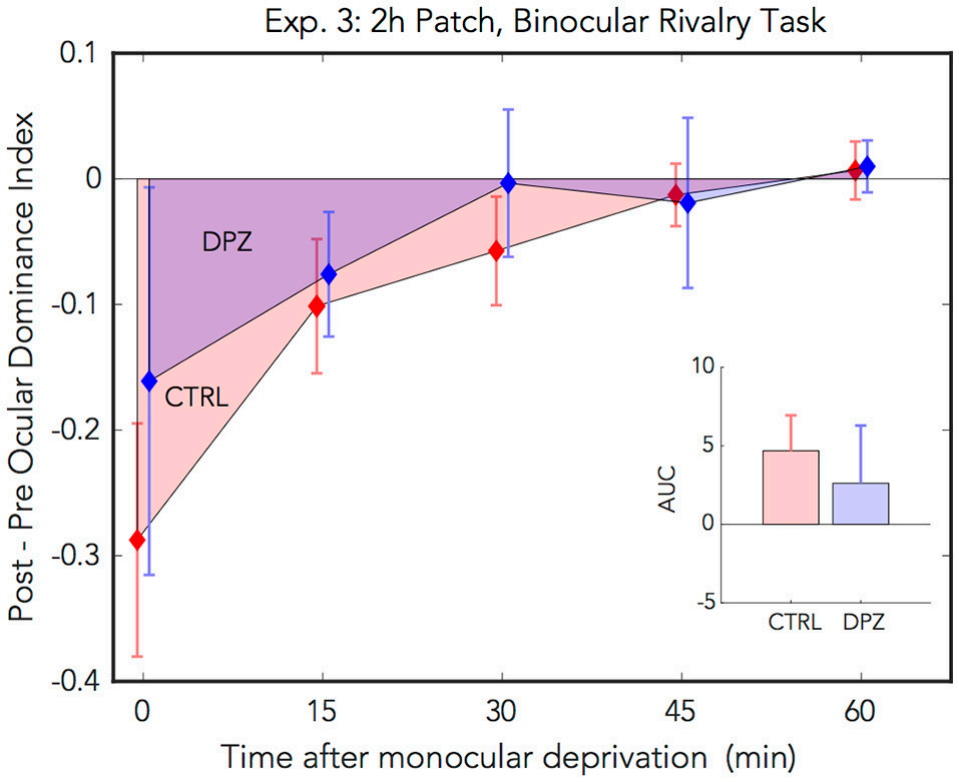
Experiment 3: The effect of donepezil on the shift in ocular dominance that occurs after 2 h of monocular deprivation, measured by binocular rivalry. Donepezil reduces the shift from baseline perceptual eye dominance relative to placebo control. *N* = 6. Red and blue diamonds indicate the mean difference from baseline OD ratio in described in Equation (4) for control and DPZ conditions, respectively. Errorbars are bootstrapped SEMs.

In addition, we performed a two-factor (session × time) repeated measures ANOVA (see Table 3 for statistics) on the post-baseline ODIs computed for measurements taken at 0, 15, 30, 45, and 60 min after removing the patch. The results of this analysis yielded a significant main effect of time [Wilks' lambda = 0.41, *F*_(4, 20)_ = 3.32, *p* < 0.05, $\eta_{p}^{2}$ = 0.40], however neither the effect of session nor the interaction term were significant (ps > 0.05) (Figure 4). While the mean shift in perceptual eye dominance across all time points was greater in the placebo control condition (*M* = −0.09, 95% CI: [−0.20, 0.02], than in the donepezil condition (*M* = −0.05, 95% CI: [−0.23, 0.13]), the lack of a significant main effect of session indicates that any observed differences between the two experimental conditions in this experiment constitute a weak effect. In addition, a two-tailed paired Wilcoxon signed-rank test on the mean ranks of the areas under the curves generated by the post-baseline ODIs in each condition did not yield a significant difference for this experiment (*p* > 0.05).

The results from our binocular rivalry experiment are less conclusive than those in the phase combination experiments, possibly due to technical limitations of our implementation of the binocular rivalry task. Although the effects observed in this experiment are weak, they nevertheless trend in the same direction as the previous experiments, namely that donepezil reduces the overall magnitude of the shift in perceptual eye dominance that occurs after temporary monocular patching.

## 4. Discussion

We conducted three experiments to investigate whether cholinergic enhancement via the AChEI donepezil could enhance the short-term perceptual eye dominance plasticity induced by 2 h of monocular patching. In Experiment 1, we used a binocular phase combination task and found that donepezil decreases the magnitude of the shift in perceptual eye dominance induced by 2 h of monocular deprivation relative to control. Importantly, donepezil also appeared to reduce the amount of time for which perceptual eye dominance was shifted. In Experiment 2, we reduced the patching duration to 1 h. We found that donepezil reduced the magnitude and duration of the shift here as well. Finally, we assessed whether the effects we observed using the binocular phase combination task were also seen using different measure of perceptual eye dominance, binocular rivalry. Our binocular rivalry result demonstrated that the magnitude of the shift in perceptual eye balance in favor of the deprived eye was reduced with donepezil compared to the placebo control. These findings agreed with that from Experiments 1 and 2. Donepezil appeared to reduce the effect of 1 and 2 h of monocular deprivation, while the effect of treatment in the control condition was significant relative to baseline.

Our study was motivated by recent findings regarding the role of cholinergic potentiation in adult visual plasticity. Specifically, repeated days of cholinergic enhancement has been shown to improve visual perceptual learning for a number of tasks in observers with normal vision (Rokem and Silver, 2010; Kang et al., 2014; Chamoun et al., 2017a), suggesting a central role of the neurotransmitter in modulating plasticity processes. In the rat, cholinergic potentiation also improves visual recovery (Chamoun et al., 2017b) and visual processing (Soma et al., 2013; Kang et al., 2015; Chamoun et al., 2016), due, in part, to enhancing the responsiveness of visual neurons to their tuned stimuli. Based on these findings, we expected a reinforcement of the shift in perceptual eye dominance in favor of the deprived eye. However, the present findings indicate that donepezil actually reduces the expected gain of the deprived eye over the non-deprived eye relative to placebo control.

There are many possible mechanisms by which ACh enhancement could cause the results we observed in our study. First, consider perceptual eye dominance as an emergent property of an aggregate population of binocular cells tuned to weighted monocular inputs. The strength of a monocular signal influencing the bias of a specific binocular pyramidal neuron is determined by three main factors: (1) the gain of thalamocortical input from a particular eye, the (2) presynaptic inhibition of the contralateral eye induced by either GABAergic interneurons or recurrent connections, or (3) long-range corticocortical projections. Changes in any or all of these three factors would result in a different perceptual eye dominance profile. Due to the presence of nicotinic and muscarinic receptors on thalamocortical fibers, inhibitory neurons and pyramidal cells, ACh is likely to influence every level of binocular summation (Groleau et al., 2015).

Notably, ACh has been shown to enhance feedforward inputs to cortex while also suppressing lateral connections within the cortex (Disney et al., 2007, 2012). Other studies report ACh-induced increases in cortical excitation as well (Hasselmo and Bower, 1992; Gil et al., 1997; Thiele, 2013; Groleau et al., 2014). As cholinergic receptors are located at every level of the cortical circuitry (Groleau et al., 2015; van Kempen et al., 2017), it is clear that ACh plays a crucial role in modulating the excitatory/inhibitory balance. We speculate that cholinergic potentiation might actually enhance feedforward thalamocortical contrast-gain, facilitating the deprived-eye's signal while simultaneously reducing the patching-induced inhibition of the non-deprived eye, causing an overall reduction in the ocular dominance shift as we observed in our study. It is likely that AChEIs affect monocular responses at the level of the lateral geniculate nucleus (which is also highly cholinoceptive), modulating monocular signal to the visual cortex. Due to the differential role of ACh in subcortical and intracortical circuits, the net reduction of the shift we observe after administration of donepezil is compatible with the idea that reduced GABAergic inhibition in early visual cortex is partially responsible for the shift in perceptual eye dominance induced by patching (Lunghi et al., 2015b).

Furthermore, it is possible that higher doses of AChEI for multiple days would have a different effect from the results we report in this article. Although the dose we administered has been shown to be effective in enhancing neural plasticity associated with perceptual learning and other aspects of visual perception in other studies (Rokem and Silver, 2010, 2013; Chamoun et al., 2016, 2017a), these studies provided multiple days of cholinergic enhancement while the present study only provided a single dose. This possibility has been called into question due to findings from a recent study (Chung et al., 2017) which reported that multiple administrations of donepezil blocked perceptual learning of a crowding task in adult human amblyopes relative to a placebo control. The finding from this study mirrors that from our own—cholinergic potentiation can reduce certain aspects of adult visual plasticity.

It may also be worthwhile to consider ACh's role in reinstating juvenile OD plasticity as a factor in our results. A previous study (Morishita et al., 2010) examining the effect of extended (30 days) MD on ocular dominance plasticity in mice found that ACh reinstates classical OD plasticity where the non-deprived eye strengthens its relative contribution to binocular vision. It is possible that our conclusions are not in conflict with the findings of this animal study. The short-term perceptual eye dominance plasticity investigated in the present study causes a shift in favor of the deprived eye. It is likely that the mechanism underlying this type of temporary visual plasticity is categorically unique from the canonic OD plasticity evaluated in the aforementioned study. The present study demonstrated that ACh impedes the consolidation of the deprived eye's enhancement after a few hours patching, causing a net shift in favor of the *non-deprived* eye relative to the placebo control. We speculate that the failure to consolidate the deprived eye's enhancement is due, in part, to two conflicting mechanisms at play: (1) the short-term perceptual eye dominance plasticity attempting to consolidate the deprived eye's enhancement and (2) the classical juvenile OD plasticity, enhanced by ACh, attempting to augment the responsiveness of the non-deprived eye. It is possible that that an ACh-modulated enhancement of juvenile OD plasticity could account for the unexpected results of the present study.

Likewise, it is plausible that ACh-mediated effects on visual attention can be a confounding factor in our results. Cholinergic potentiation is known to play a critical role in the top-down control of attentional orienting and stimulus discrimination (Klinkenberg et al., 2011; Groleau et al., 2015). While the binocular phase combination task is robust to changes in attentional control since it does not require substantial stimulus discrimination or attentional orienting, it is now widely agreed that binocular rivalry is highly influenced by attention (see Dieter et al., 2016 for a review). While our binocular rivalry results are consistent with those reported in our binocular phase combination results, it remains an open question whether short-term monocular deprivation alters fused or eye-specific attentional dynamics, and yet another question is whether cholinergic enhancement affects these dynamics.

Our finding that donepezil reduces the magnitude and duration of the perceptual eye dominance plasticity induced by a few hours of monocular patching contributes to the growing evidence that cholinergic potentiation enhances some aspects of adult visual function and plasticity at the expense of others. Further work is necessary to determine whether the short-term perceptual eye dominance plasticity evaluated in this study can be enhanced pharmacologically. This line of research has the dual benefit of adding to possible clinical therapies for visual disorders while also enhancing our understanding of the limitations and mechanisms of adult neural plasticity.

## Author Contributions

AB, EV, MC, and RH collaboratively conceived the project idea. AB and YS implemented the psychophysical tests. MC, PR-N, and YS conducted the experiments. YS conducted statistical analyses on the data and drafted the manuscript. AB, EV, MC, RH, and YS contributed to revising the manuscript.

### Conflict of Interest Statement

The authors declare that the research was conducted in the absence of any commercial or financial relationships that could be construed as a potential conflict of interest.

## References

[B1] Bai J. Dong X. He S. Bao M. (2017). Monocular deprivation of Fourier phase information boosts the deprived eye's dominance during interocular competition but not interocular phase combination. Neuroscience 352, 122–130. 10.1016/j.neuroscience.2017.03.053 28391010

[B2] Baldwin A. S. Hess R. F. (2018). The mechanism of short-term monocular deprivation is not simple: separate effects on parallel and cross-oriented dichoptic masking. Sci. Rep. 8:6191 10.1038/s41598-018-24584-9 29670145 PMC5906446

[B3] Bao S. Chan V. T. Merzenich M. M. (2001). Cortical remodelling induced by activity of ventral tegmental dopamine neurons. Nature 412, 79–83. 10.1038/35083586 11452310

[B4] Baroncelli L. Bonaccorsi J. Milanese M. Bonifacino T. Giribaldi F. Manno I.. (2012). Enriched experience and recovery from amblyopia in adult rats: impact of motor, social and sensory components. Neuropharmacology 62, 2387–2396. 10.1016/j.neuropharm.2012.02.010 22532989

[B5] Baroncelli L. Maffei L. Sale A. (2011). New perspectives in amblyopia therapy on adults: a critical role for the excitatory/inhibitory balance. Front. Cell. Neurosci. 5:25. 10.3389/fncel.2011.00025 22144947 PMC3223381

[B6] Bavelier D. Levi D. M. Li R. W. Dan Y. Hensch T. (2010). Removing brakes on adult brain plasticity: from molecular to behavioural interventions. J. Neurosci. 30, 14964–14971. 10.1523/JNEUROSCI.4812-10.2010 21068299 PMC2992973

[B7] Bear M. F. Singer W. (1986). Modulation of visual cortical plasticity by acetylcholine and noradrenaline. Nature 320, 172–176. 10.1038/320172a0 3005879

[B8] Benajmini Y. Hochberg Y. (1995). Controlling the false discovery rate: a practical and powerful approach to multiple testing. J. R. Stat. Soc. Ser. B 57, 289–300. 10.1111/j.2517-6161.1995.tb02031.x

[B9] Bentley P. Vuilleumier P. Thiel C. M. Driver J. Dolan R. J. (2003). Cholinergic enhancement modulates neural correlates of selective attention and emotional processing. NeuroImage 20, 58–70. 10.1016/S1053-8119(03)00302-1 14527570

[B10] Berardi N. Pizzorusso T. Maffei L. (2000). Critical periods during sensory development. Curr. Opin. Neurobiol. 10, 138–145. 10.1016/S0959-4388(99)00047-1 10679428

[B11] Binda P. Kurzawski J. Lunghi C. Biagi L. Tosetti M. Morrone M. C. (2017). Short-term monocular deprivation enhances 7T BOLD responses and reduces neural selectivity in V1. J. Vis. 17:577 10.1167/17.10.577

[B12] Brainard D. H. (1997). The psychophysics toolbox. Spatial Vis. 4, 433–436. 10.1163/156856897X00357 9176952

[B13] Chadnova E. Reynaud A. Clavagnier S. Hess R. F. (2017). Short-term monocular occlusion produces changes in ocular dominance by a reciprocal modulation of interocular inhibition. Sci. Rep. 7:41747. 10.1038/srep41747 28150723 PMC5288724

[B14] Chamoun M. Groleau M. Bhat M. Vaucher E. (2016). Dose-dependent effect of donepezil administration on long-term enhancement of visually evoked potentials and cholinergic receptor overexpression in rat visual cortex. J. Physiol. Paris 110, 65–74. 10.1016/j.jphysparis.2016.11.010 27913166

[B15] Chamoun M. Huppé-Gourgues F. Legault I. Rosa-Neto P. Dumbrava D. Faubert J.. (2017a). Cholinergic potentiation improves perceptual-cognitive training of healthy young adults in three dimensional multiple object tracking. Front. Hum. Neurosci. 11:128. 10.3389/fnhum.2017.00128 28377707 PMC5359296

[B16] Chamoun M. Sergeeva E. G. Henrich-Noack P. Jia S. Grigartzik L. Ma J.. (2017b). Cholinergic potentiation of restoration of visual function after optic nerve damage in rats. Neural Plast. 2017:6928489. 10.1155/2017/6928489 28928986 PMC5592016

[B17] Chung S. T. Li R. W. Silver M. A. Levi D. M. (2017). Donepezil does not enhance perceptual learning in adults with amblyopia: a pilot study. Front. Neurosci. 11:448 10.3389/fnins.2017.00448 28824369 PMC5545606

[B18] Conner J. M. Culberson A. Packowski C. Chiba A. A. Tuszynski M. H. (2003). Lesions of the basal forebrain cholinergic system impair task acquisition and abolish cortical plasticity associated with motor skill learning. Neuron 38, 819–829. 10.1016/S0896-6273(03)00288-5 12797965

[B19] Dieter K. C. Brascamp J. Tadin D. Blake R. (2016). Does visual attention drive the dynamics of bistable perception? Attent. Percept. Psychophys. 78, 1861–1873. 10.3758/s13414-016-1143-2 27230785 PMC5014653

[B20] Ding J. Sperling G. (2006). A gain-control theory of binocular combination. Proc. Natl. Acad. Sci. U.S.A. 103, 1141–1146. 10.1073/pnas.0509629103 16410354 PMC1347993

[B21] Disney A. A. Aoki C. Hawken M. J. (2007). Gain modulation by nicotine in macaque V1. Neuron 56, 701–713. 10.1016/j.neuron.2007.09.034 18031686 PMC2875676

[B22] Disney A. A. Aoki C. Hawken M. J. (2012). Cholinergic suppression of visual responses in primate V1 is mediated by GABAergic inhibition. J. Neurophysiol. 108, 1907–1923. 10.1152/jn.00188.2012 22786955 PMC3545006

[B23] Fagiolini M. Hensch T. K. (2000). Inhibitory threshold for critical-period activation in primary visual cortex. Nature 404, 183–186. 10.1038/35004582 10724170

[B24] Gagolewicz P. J. Dringenberg H. C. (2009). Selective potentiation of crossed vs. uncrossed inputs from lateral geniculate nucleus to visual cortex by the basal forebrain: potential facilitation of rodent binocularity. Neurosci. Lett. 463, 130–134. 10.1016/j.neulet.2009.07.052 19631720

[B25] Gil Z. Connors B. W. Amitai Y. (1997). Differential regulation of neocortical synapses by activity and neuromodulators. Neuron 19, 679–686. 9331357 10.1016/s0896-6273(00)80380-3

[B26] Gilbert C. D. Li W. (2012). Adult visual cortical plasticity. Neuron 75, 250–264. 10.1016/j.neuron.2012.06.030 22841310 PMC3408614

[B27] Gratton C. Yousef S. Aarts E. Wallace D. L. D'Esposito M. Silver M. A. (2017). Cholinergic, but not dopaminergic or noradrenergic, enhancement sharpens visual spatial perception in humans. J. Neurosci. 37, 4405–4415. 10.1523/JNEUROSCI.2405-16.2017 28336568 PMC5413181

[B28] Groleau M. Kang J. I. Huppé-Gourgues F. Vaucher E. (2015). Distribution and effects of the muscarinic receptor subtypes in the primary visual cortex. Front. Synapt. Neurosci. 7:10. 10.3389/fnsyn.2015.00010 26150786 PMC4472999

[B29] Groleau M. Nguyen H. N. Vanni M. P. Huppé-Gourgues F. Casanova C. Vaucher E. (2014). Impaired functional organization in the visual cortex of muscarinic receptor knock-out mice. NeuroImage 98, 233–242. 10.1016/j.neuroimage.2014.05.016 24837499

[B30] Hasselmo M. E. Bower J. M. (1992). Cholinergic suppression specific to intrinsic not afferent fiber synapses in rat piriform (olfactory) cortex. J. Neurophysiol. 67, 1222–1229. 10.1152/jn.1992.67.5.1222 1597708

[B31] Hess R. F. Zhou J. Clavagnier S. (2013). Short-term monocular deprivation strengthens the patched eye's contribution to binocular combination. J. Vis. 13:12. 10.1167/13.5.12 23599416

[B32] Huang C.-B. Zhou J. Lu Z.-L. Feng L. Zhou Y. (2009). Binocular combination in anisometropic amblyopia. J. Vis. 9, 17.1–17.16. 10.1167/9.3.17 19757956 PMC2861488

[B33] Hubel D. H. Wiesel T. N. (1970). The period of susceptibility to the physiological effects of unilateral eye closure in kittens. J. Physiol. 206, 419–436. 10.1113/jphysiol.1970.sp009022 5498493 PMC1348655

[B34] Kang J. I. Huppé-Gourgues F. Vaucher E. (2014). Boosting visual cortex function and plasticity with acetylcholine to enhance visual perception. Front. Syst. Neurosci. 8:172. 10.3389/fnsys.2014.00172 25278848 PMC4167004

[B35] Kang J. I. Huppé-Gourgues F. Vaucher E. (2015). Pharmacological mechanisms of cortical enhancement induced by the repetitive pairing of visual/cholinergic stimulation. PLoS ONE 10:e141663. 10.1371/journal.pone.0141663 26513575 PMC4626033

[B36] Kasamatsu T. Ohashi T. Imamura K. (1991). Lithium reduces ocular dominance plasticity in kitten visual cortex. Brain Res. 558, 157–162. 10.1016/0006-8993(91)90735-E 1933378

[B37] Kim H.-W. Kim C.-Y. Blake R. (2017). Monocular perceptual deprivation from interocular suppression temporarily imbalances ocular dominance. Curr. Biol. 27, 884–889. 10.1016/j.cub.2017.01.063 28262490

[B38] Klink P. C. Brascamp J. W. Blake R. Van Wezel R. J. A. (2010). Experience-driven plasticity in binocular vision. Curr. Biol. 20, 1464–1469. 10.1016/j.cub.2010.06.057 20674360 PMC2926173

[B39] Klinkenberg I. Sambeth A. Blokland A. (2011). Acetylcholine and attention. Behav. Brain Res. 221, 430–442. 10.1016/j.bbr.2010.11.033 21108972

[B40] Lunghi C. Berchicci M. Morrone M. C. Di Russo F. (2015a). Short-term monocular deprivation alters early components of visual evoked potentials. J. Physiol. 593, 4361–4372. 10.1113/JP270950 26119530 PMC4594246

[B41] Lunghi C. Burr D. C. Morrone C. (2011). Brief periods of monocular deprivation disrupt ocular balance in human adult visual cortex. Curr. Biol. 21, R538–R539. 10.1016/j.cub.2011.06.004 21783029

[B42] Lunghi C. Emir U. E. Morrone M. C. Bridge H. (2015b). Short-term monocular deprivation alters GABA in the adult human visual cortex supplemental figures and legends. Curr. Biol. 25, 1496–1501. 10.1016/j.cub.2015.04.021 26004760 PMC5040500

[B43] Lunghi C. Morrone M. C. Secci J. Caputo R. (2016). Binocular rivalry measured 2 hours after occlusion therapy predicts the recovery rate of the amblyopic eye in anisometropic children. Invest. Ophthalmol. Vis. Sci. 57, 1537–1546. 10.1167/iovs.15-18419 27046118 PMC4909145

[B44] Maya Vetencourt J. F. Sale A. Viegi A. Baroncelli L. Pasquale R. D. Leary O. F. O. (2008). The antidepressant fluoxetine restores plasticity in the adult visual cortex. Science 510, 385–389. 10.1126/science.1150516 18420937

[B45] Miles W. R. (1930). Ocular dominance in human adults. J. Gen. Psychol. 4, 412–430.

[B46] Morishita H. Miwa J. M. Heintz N. Hensch T. K. (2010). Lynx1, a cholinergic brake, limits plasticity in adult visual cortex. Science 330, 1238–1241. 10.1126/science.1195320 21071629 PMC3387538

[B47] O'Shea R. P. (2017). Adult neuroplasticity: working one eye gives an advantage to the other. Curr. Biol. 27, R230–R231. 10.1016/j.cub.2017.02.021 28324741

[B48] Pelli D. G. (1997). Pixel independence: measuring spatial interactions on a CRT display. Spatial Vis. 10, 443–446. 10.1163/156856897X00375 9176954

[B49] Prvulovic D. Schneider B. (2014). Pharmacokinetic and pharmacodynamic evaluation of donepezil for the treatment of Alzheimer's disease. Expert Opin. Drug Metab. Toxicol. 10, 1039–1050. 10.1517/17425255.2014.915028 24785550

[B50] Rogers S. L. Friedhoff L. T. (1998). Pharmacokinetic and pharmacodynamic profile of donepezil HCl following single oral doses. Br. J. Clin. Pharmacol. 46, 7–12. 10.1046/j.1365-2125.1998.0460s1007.x 9839758 PMC1873812

[B51] Rokem A. Silver M. A. (2010). Cholinergic enhancement augments magnitude and specificity of visual perceptual learning in healthy humans. Curr. Biol. 20, 1723–1728. 10.1016/j.cub.2010.08.027 20850321 PMC2953574

[B52] Rokem A. Silver M. A. (2013). The benefits of cholinergic enhancement during perceptual learning are long-lasting. Front. Comput. Neurosci. 7:66. 10.3389/fncom.2013.00066 23755006 PMC3665931

[B53] Silver M. A. Shenhav A. D'Esposito M. (2008). Cholinergic enhancement reduces spatial spread of visual responses in human early visual cortex. Neuron 60, 904–914. 10.1016/j.neuron.2008.09.038 19081383 PMC2640421

[B54] Soma S. Suematsu N. Shimegi S. (2013). Cholinesterase inhibitor, donepezil, improves visual contrast detectability in freely behaving rats. Behav. Brain Res. 256, 362–367. 10.1016/j.bbr.2013.08.022 23994545

[B55] Thiele A. (2013). Muscarinic signaling in the brain. Annu. Rev. Neurosci. 36, 271–294. 10.1146/annurev-neuro-062012-170433 23841840

[B56] Tso D. Miller R. Begum M. (2017). Neuronal responses underlying shifts in interocular balance induced by short-term deprivation in adult macaque visual cortex. J. Vis. 17:576 10.1167/17.10.576

[B57] van Kempen J. Panzeri S. Thiele A. (2017). Cholinergic control of information coding. Trends Neurosci. 40, 522–524. 10.1016/j.tins.2017.06.006 28693847

[B58] Weinberger N. M. (2007). Auditory associative memory and representational plasticity in the primary auditory cortex. Hear. Res. 229, 54–68. 10.1016/j.heares.2007.01.004 17344002 PMC2693954

[B59] Wiesel T. N. (1982). The postnatal development of the visual cortex and the influence of environment. Biosci. Rep. 2, 351–377. 10.1007/BF01119299 7049262

[B60] Zhou J. Baker D. H. Simard M. Saint-Amour D. Hess R. F. (2015). Short-term monocular patching boosts the patched eye's response in visual cortex. Restor. Neurol. Neurosci. 33, 381–387. 10.3233/RNN-140472 26410580 PMC4923712

[B61] Zhou J. Thompson B. Hess R. F. (2013). A new form of rapid binocular plasticity in adult with amblyopia. Sci. Rep. 3:2638. 10.1038/srep02638 24026421 PMC3770967

[B62] Zhou Y. H. Gao J. B. White K. D. Merk I. Yao K. (2004). Perceptual dominance time distributions in multistable visual perception. Biol. Cybern. 90, 256–263. 10.1007/s00422-004-0472-8 15085344

[B63] Zucker R. S. Regehr W. G. (2002). Short-term synaptic plasticity. Annu. Rev. Physiol. 64, 355–405. 10.1146/annurev.physiol.64.092501.114547 11826273

